# Multi‐Omics Reveal the Metabolic Changes in Cumulus Cells During Aging

**DOI:** 10.1111/cpr.70014

**Published:** 2025-03-05

**Authors:** Liangyue Shi, Hengjie Wang, Shuai Zhu, Minjian Chen, Xuejiang Guo, Qiang Wang, Ling Gu

**Affiliations:** ^1^ College of Animal Science & Technology Nanjing Agricultural University Nanjing China; ^2^ State Key Laboratory of Reproductive Medicine and Offspring Health Nanjing Medical University Nanjing China; ^3^ Changzhou Maternity and Child Health Care Hospital, Changzhou Medical Center Nanjing Medical University Nanjing China

**Keywords:** aging, cumulus cells, metabolomics, oocyte, proteomic

## Abstract

Maternal age has been reported to impair oocyte quality. However, the molecular mechanisms underlying the age‐related decrease in oocyte competence remain poorly understood. Cumulus cells establish direct contact with the oocyte through gap junctions, facilitating the provision of crucial nutrients necessary for oocyte development. In this study, we obtained the proteomic and metabolomic profiles of cumulus cells from both young and old mice. We found that fatty acid beta‐oxidation and nucleotide metabolism, markedly active in aged cumulus cells, may serve as a compensatory mechanism for energy provision. Tryptophan undergoes two principal metabolic pathways, including the serotonin (5‐HT) synthesis and kynurenine catabolism. Notably, we discovered that kynurenine catabolism is reduced in aged cumulus cells compared to young cells, whereas 5‐HT synthesis exhibited a significant decrease. Furthermore, the supplement of 5‐HT during cumulus‐oocyte complexes (COCs) culture significantly ameliorated the metabolic dysfunction and meiotic defects in old oocytes. In sum, our data provide a comprehensive multiple omics resource, offering potential insights for improving oocyte quality and promoting fertility in aged females.

## Graphical abstract

Fatty acid β‐oxidation was elevated in cumulus cells from older mice. Tryptophan metabolism in aged cumulus cells was active. Supplementing with 5‐HT could mitigate aging damage in oocytes.

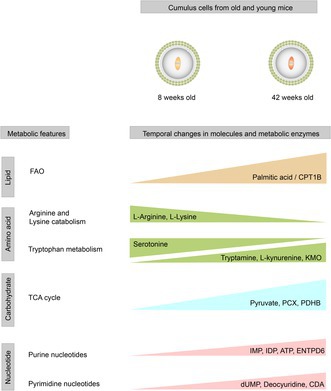

## 1 Introduction

The mammalian ovary is a dynamic reproductive endocrine organ responsible for producing the ovum and providing sex steroids required for female fertility and quality of life [1]. Unlike sperm, the quantity of follicle reserve is predetermined before birth, which means that germ cells in ovaries cannot replenish it and each subsequent ovulation after puberty signifies a reduction in the follicles. Mammalian follicles consist of oocytes, theca cells and granulosa cells. Primordial follicles are characterised by a single layer of flattened granulosa cells surrounding the oocyte. However, the majority of primordial follicles tend to progress towards atresia follicles, unless they undergo transformation into primary follicles. Primary follicles exhibit a single to multiple layers of cuboidal granulosa cells without the presence of theca cells [2, 3]. As the follicles gradually develop and enlarge, granulosa cells change into multiple layers and cuboidal, theca cells appear in secondary follicles [4]. During follicle maturation, granulosa cells surrounding a single oocyte differentiate into cumulus cells [5]. Gap junctions form between oocytes and cumulus cells, facilitating the transfer of energy sources and communication between them [6, 7].

The female fertility and reproductive lifespan decline dramatically with age, especially after the mid‐30s [8]. Previous studies have shown that approximately 10% of women under 34 years of age are unable to conceive naturally; however, this proportion astonishingly rises to 87% over the age of 45 years [9]. Advanced maternal age may be at an increased risk of infertility, spontaneous abortion, foetal death and congenital anomalies [10, 11]. One of the reasons for this situation is the decline of oocyte quality [12]. It has been shown that maternal age influences the meiotic progress, oxidative stress and particularly genomic stability. For example, we identified a substantial reduction in SIRT2 in oocytes from old mice, which not only increased meiotic defects and incidence of aneuploidy but also induced mitochondrial dysfunction, ultimately resulting in impaired oocyte quality [13]. The intercellular interactions between cumulus cells and oocytes are seen as an important factor in oocyte maturation, and cumulus cells play an essential nurturing role in supporting oocyte development [14, 15, 16, 17]. The relationship between oocytes and cumulus cells provides several key benefits for preserving the genomic stability and integrity of the oocyte, which is essential for reproductive success. Firstly, cumulus cells sense environmental factors and shield oocytes from external stimulation, such as glutathione (GSH), an important tripeptide thiol antioxidant produced by cumulus cells to defend against cellular oxidative damage. In addition, cumulus cells provide the essential metabolites to buffer metabolic defects in the oocyte. For instance, cumulus cells convert glucose to pyruvate via glycolysis, followed by pyruvate oxidation in oocyte mitochondria to produce energy for development and maturation. Therefore, the oocyte relies heavily on cumulus cells to maintain normal development and function; however, whether aging affects oocyte quality by interfering with the metabolic pattern in cumulus cells requires deeper exploration.

In the present study, we acquired the metabolic profile of cumulus cells from old and young mice. Simultaneously, quantitative proteomic analysis enabled the identification of the key enzymes responsible for the observed metabolic changes in cumulus cells. Establishing metabolic networks may hold promise in enhancing our understanding of the regulatory mechanisms influencing aged oocytes.

## 2 Results

### 2.1 Proteomic and Metabolomic Profiling of Cumulus Cells From Young and Old Mice

**FIGURE 1:**
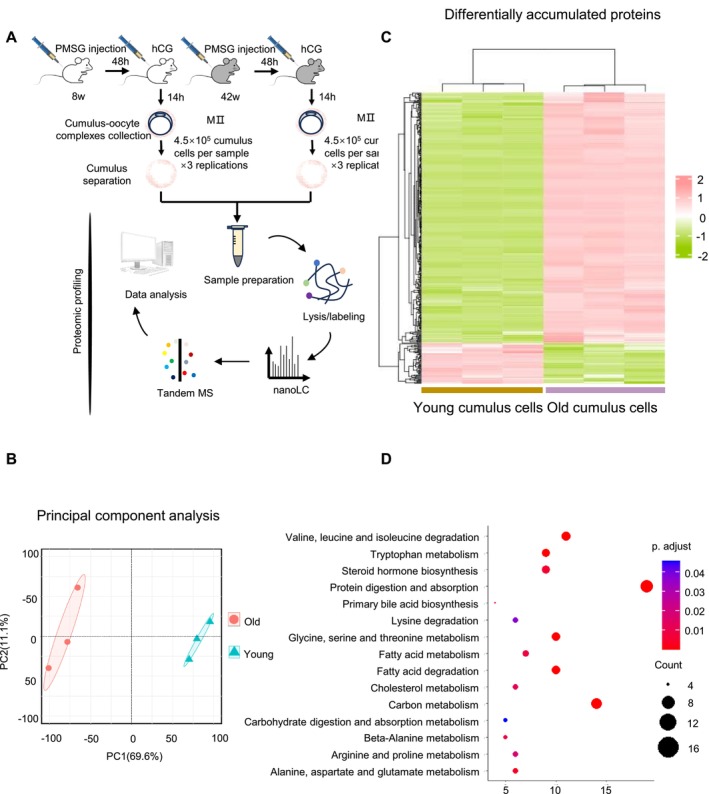
Proteomic profiling of cumulus cells (A) Schematic overview of the workflow for proteome profiling in cumulus cells. (B) Principal component analysis of cumulus cell heterogeneity. (C) Heatmap of 415 differentially expressed proteins among young and old cumulus cells. (D) Bubble chart of enriched KEGG pathway terms for all differentially accumulated proteins in old cumulus cells. The complete proteomics data are available in Table S1.

Previous studies primarily focused on oocytes in aging animals, but there has been few research in cumulus cells. In our present study, we conducted quantitative proteomic analysis of cumulus cells (three repeats and 4.5 × 10^5^ cumulus cells separated from 375 MII cumulus‐oocyte complexes [COCs] per sample) from young and old mice (Figure 1A). In total, more than 9000 proteins were detected, including 415 differentially accumulated proteins (false discovery rate [FDR] = 0.05). Principal component analysis was utilised to evaluate the samples in different groups (Figure 1B). The differentially accumulated proteins of the two groups are displayed in Figure 1C. Moreover, Kyoto Encyclopedia of Genes and Genomes (KEGG) enrichment analysis revealed significant enrichment in categories associated with metabolic pathways (Figure 1D).

**FIGURE 2:**
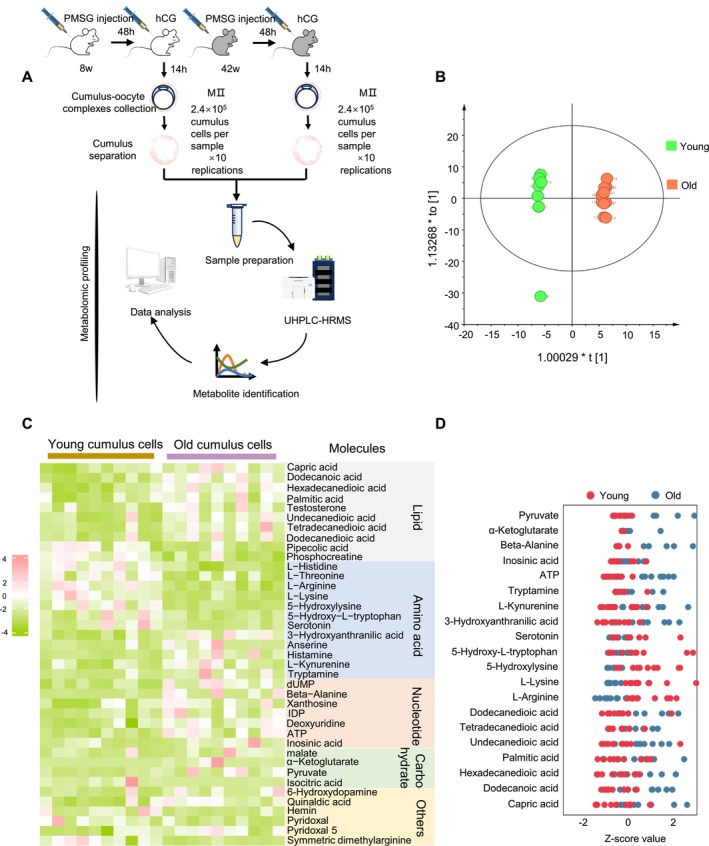
Metabolomic profiling of cumulus cells (A) Collection of cumulus cells in vivo from young and old mice and workflow for UPLC/MS‐based metabolome profiling on cumulus cells. (B) OPLS‐DA score plot for metabolomic datasets clearly distinguishes cumulus cell samples from two groups. (C) Heatmap visualising relative abundance of differential metabolites in young and old cumulus cells, classified by metabolic pathway. (D) *Z*‐score plots of 20 representative differential metabolites in cumulus cells from old and young mice. The complete metabolomic data are available in Table S2.

Metabolites serve as either products or direct substrates of metabolic enzymes. Changes in metabolite levels typically coincide with alterations in related enzymes. To verify the relationship between differential metabolites and metabolic enzymes, we collected a substantial number of cumulus cells from COCs from old and young mice (2.4 × 10^5^ cumulus cells separated from 200 MII COCs per sample). All samples were analysed by ultra‐high‐performance liquid chromatography‐tandem high‐resolution mass spectrometry for the intracellular metabolome (Figure 2A). A total of 71 differential metabolites were identified based on *p* value (*p* < 0.05) and the variable importance in projection (VIP) value (VIP > 1). Robust orthogonal partial least squares‐discriminant analysis (OPLS‐DA) revealed distinct clusters between the two groups (Figure 2B). The differential metabolites of the two groups are displayed in Figure 2C. In general, compared with the young group, the levels of most metabolites involved in carbohydrates, lipids and nucleotides were increased in cumulus cells from the old group, whereas many amino acid metabolites indicated a down‐regulated trend (Figure 2C,D). The integration of metabolomic and proteomic data was crucial in characterising the metabolic features present in cumulus cells from ageing mice.

### 2.2 Lipid Metabolism in Cumulus Cells From Old Mice

**FIGURE 3:**
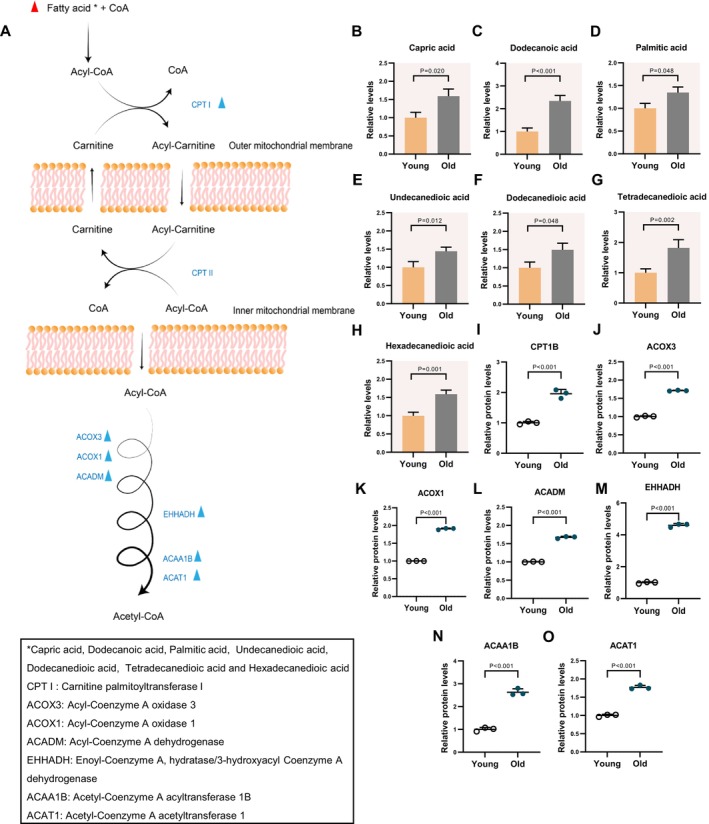
Increased fatty acid beta‐oxidation in aged cumulus cells (A) Schematic diagram of fatty acid beta‐oxidation. Metabolites with increased levels from old mice are indicated by bold red triangles. Changes in differential metabolic enzymes are indicated by blue triangles. (B–H) Relative levels of metabolites related to fatty acid oxidation in young and aged cumulus cells. (I–O) Relative abundance of the representative enzymes involved in fatty acid beta‐oxidation. Error bars, SEM. Student's *t‐* test was used for statistical analysis in all panels, comparing to young cumulus cells. n.s., not significant.

Lipid metabolism of mammalian cumulus cells plays a crucial role in the growth and maturation of oocytes [18]. It has been suggested that lipid metabolism is closely related to energy homeostasis, as depicted in Figure 3A. Nevertheless, the impact of aging on cumulus cells remains to be fully understood. In our study, we observed an elevation in the levels of medium‐and long‐chain fatty acids associated with beta‐oxidation in aged cumulus cells.

#### 2.2.1 Elevated Fatty Acid β‐Oxidation in Cumulus Cells From Old Mice

Due to its high energy density, adenosine triphosphate production from fatty acid is higher than that from glucose [19, 20]. Beta‐oxidation of fatty acid is one of the key processes that decomposes fatty acids into energy, commonly observed in the energy supply of the follicular environment. Fatty acid beta‐oxidation, a crucial process occurring in mitochondria, involves the breakdown of fatty acids to generate energy. Upon entry into the outer mitochondrial membrane, long‐chain acyl‐CoA interacts with carnitine, resulting in the formation of acylcarnitine, a process facilitated by the enzyme carnitine palmitoyl transferase 1 (CPT1). Subsequently, acylcarnitine is transported through carnitine‐acylcarnitine translocase across the inner mitochondrial membrane, where acylcarnitine is converted into fatty acyl‐CoA by carnitine palmitoyl transferase II (CPT2) to undergo beta‐oxidation in the mitochondrial matrix [21, 22, 23]. Our metabolomic profiling identified elevated levels of seven long‐chain fatty acids: capric acid, dodecanoic acid, palmitic acid, undecanedioic acid, dodecanedioic acid, tetradecanedioic acid and hexadecanedioic acid (Figure 3B–H). Notably, dodecanoic acid and hexadecanedioic acid levels in cumulus cells from aged mice were significantly higher compared to those from younger mice. Additionally, we also observed a significant increase in the levels of seven enzymes involved in fatty acid beta‐oxidation (i.e., CPT1B, ACOX1, ACOX3, ACADM, EHHADH, ACAA1B and ACAT1) in cumulus cells from aged mice (Figure 3I–O), indicative of the enhancement of the fatty acid beta‐oxidation pathway in cumulus cells from aged mice.

### 2.3 Amino Acid Metabolism in Cumulus Cells From Old Mice

**FIGURE 4:**
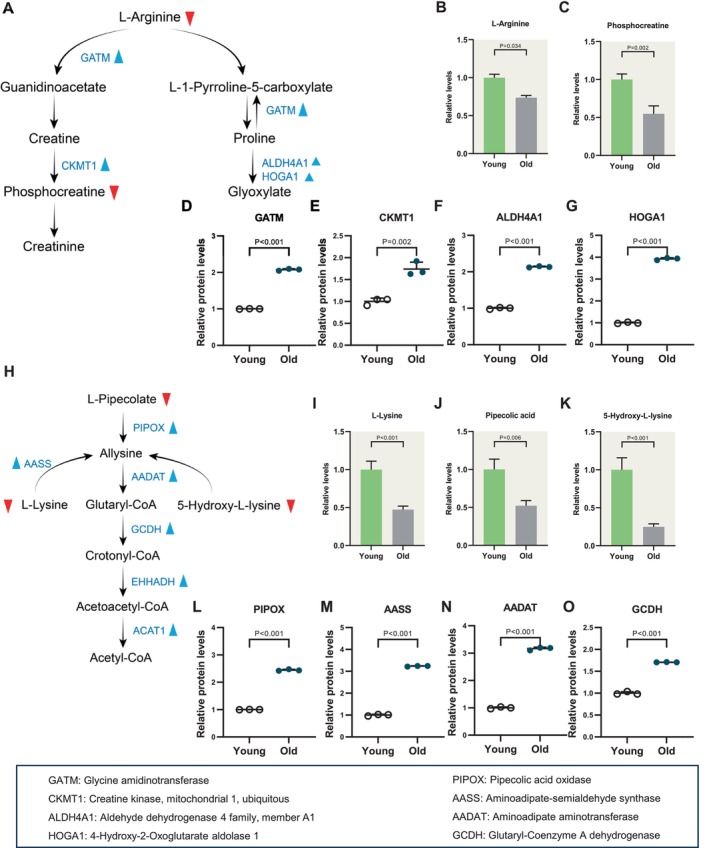
Increased arginine and lysine catabolism in aged cumulus cells (A) Schematic diagram of arginine metabolism. Metabolites with decreased levels are indicated by bold red triangles. Changes in differential metabolic enzymes are indicated by blue triangles. (B and C) Relative levels of metabolites related to arginine in young and old cumulus cells. (D–G) Relative abundance of the representative enzymes involved in arginine metabolism. (H) Schematic diagram of lysine degradation. Decreased metabolites in old cumulus cells are indicated by bold red triangles. Changes in differential metabolic enzymes are indicated by blue triangles. (I–K) Relative levels of metabolites related to lysine in young and old cumulus cells. (L–O) Relative abundance of the representative enzymes involved in lysine degradation. Error bars, SEM. Student's *t*‐test was used for statistical analysis in all panels, comparing to young group cumulus cells. n.s., not significant.

Protein synthesis and decomposition constitute indispensable physiological processes for biological functions, contributing significantly to growth and development [24]. Despite their vital importance, there remains a limited understanding of the metabolic dynamics of amino acids in cumulus cells from old mice. Through a comprehensive analysis of the cumulus cell metabolome and proteome, we elucidated distinctive features of amino acid metabolism during aging. Notably, we observed a reduction in the abundance of most amino acids in cumulus cells from aged mice (e.g., arginine, lysine, threonine and histidine) (Figure 4A,H, S1 and S2). In contrast, some metabolites were elevated in the aged group (e.g., kynurenine, tryptamine and anserine) (Figure 6A). Overall, such diversity of metabolic trends in aged cumulus cells reflects the complex regulation of amino acid metabolism.

#### 2.3.1 Increased Catabolism of Arginine and Lysine in Aged Cumulus Cells

Previous studies have demonstrated the favourable effects of arginine on cumulus cell integrity, contributing to the proliferation of cumulus cells [25]. Moreover, arginine serves as the substrate for the synthesis of nitric oxide (NO) in vivo, a factor known to facilitate the maintenance of high oocyte quality [26, 27]. In our study, we observed that five metabolites (arginine, phosphocreatine, L‐pipecolate, lysine and 5‐hydrox‐L‐lysine) in arginine metabolism and lysine degradation were reduced in aged cumulus cells (Figure 4B,C,I–K). Consistent with this observation, proteomic analyses revealed the elevated levels of enzymes associated with lysine degradation and the creatine pathway in cumulus cells (i.e., GATM, CKMT1, AASS, AADAT, GCDH, EHHADH and ACAT1) (Figure 4D–G,L–O). The primary metabolic function of creatine involves the generation of phosphocreatine through creatine kinase and phosphate groups, thereby contributing to ATP regeneration. Together, these findings indicate an augmented utilisation of arginine and lysine catabolism in aged cumulus cells, potentially serving as a crucial energy source to maintain physiological function.

#### 2.3.2 Increased Branched‐Chain Amino Acids Degradation in Aged Cumulus Cells

**FIGURE 5:**
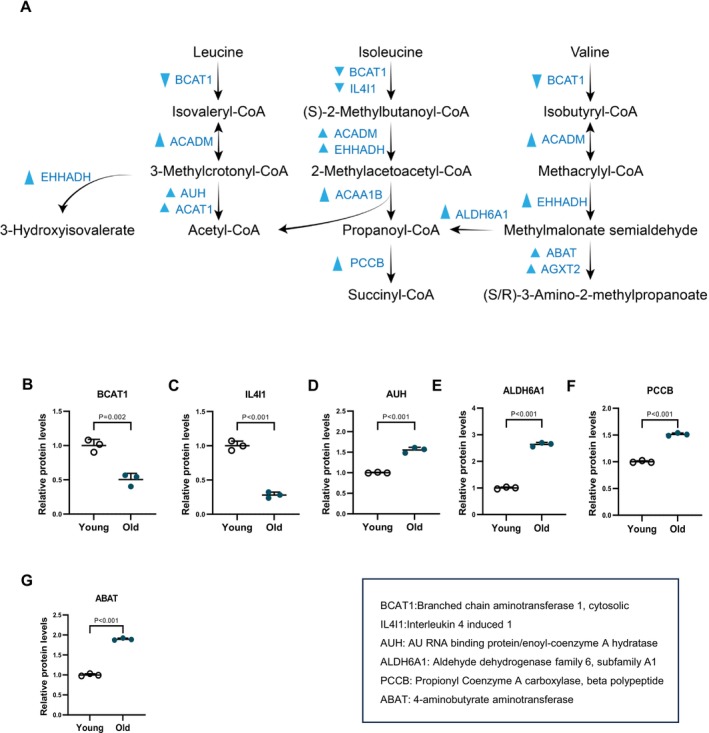
Elevated branched‐chain amino acids degradation during aging process (A) Schematic diagram of branched‐chain amino acids degradation. Changes in differential metabolic enzymes are indicated by blue triangles. (B–G) Relative abundance of the representative enzymes involved in branch‐chain amino acid degradation. Error bars, SEM. Student's *t‐*test was used for statistical analysis in all panels, comparing to young cumulus cells. n.s., not significant.

The branched‐chain amino acids (BCAAs), valine, leucine and isoleucine are essential amino acids, which have been studied in a number of disorders, including cancer, burn injury and liver cirrhosis. BCAA supplementation has been thought to promote anabolic pathways, thereby potentially preventing or treating signs of hepatic encephalopathy, facilitating wound healing, and stimulating insulin production [28]. In the present study, we found that 9 out of 11 enzymes involved in the valine, leucine and isoleucine degradation (i.e., ACADM, EHHADH, AUH, ACAT1, ACAA1B, ALDH6A1, PCCB, ABAT, AGXT2) were elevated in aged cumulus cells (Figure 5B–H). Nonetheless, while the expression of multiple protein levels was upregulated, no significant difference in metabolites was detected (Figure 5A). We propose that increased utilisation of valine, leucine and isoleucine may be related to energy supply in aged cumulus cells.

#### 2.3.3 Active Tryptophan Metabolism in Aged Cumulus Cells

**FIGURE 6:**
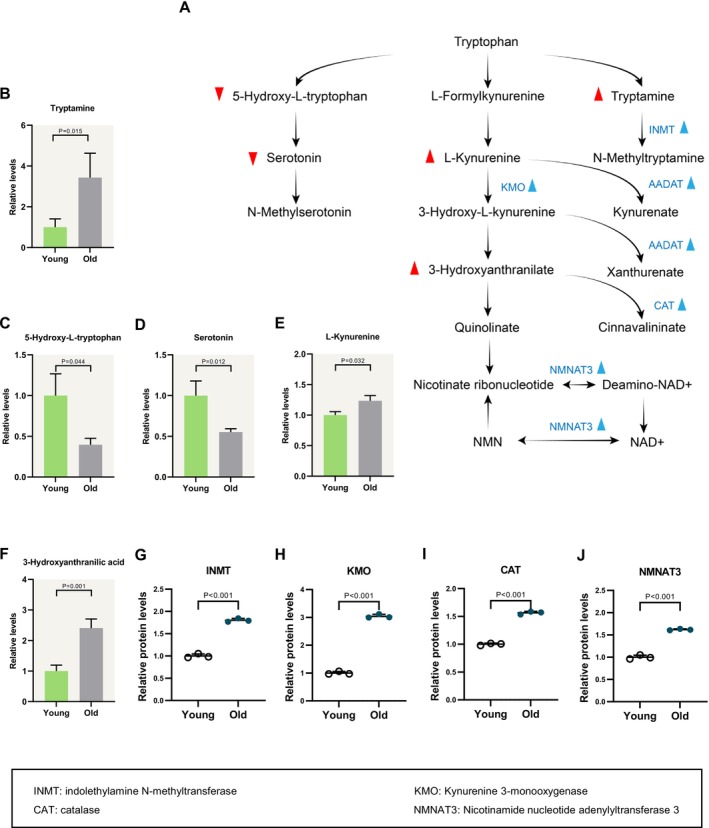
Active tryptophan metabolism during aging process (A) Schematic diagram of tryptophan metabolism. Differential metabolites in cumulus cells are indicated by bold red triangles. Changes in differential metabolic enzymes are indicated by blue triangles. (B–F) Relative levels of metabolites related to tryptophan in young and old cumulus cells. (G–J) Relative abundance of the representative enzymes involved in tryptophan metabolism. Error bars, SEM. Student's *t*‐test was used for statistical analysis in all panels, comparing to young cumulus cells. n.s., not significant.

Tryptophan undergoes two principal metabolic pathways within the body. One pathway involves the synthesis of serotonin through oxidative decarboxylation, while the other results in the production of CO_2_ and H_2_O through kynurenine catabolism [29]. The products of the tryptophan metabolism pathway play roles in various aspects. L‐formylkynurenine is the first rate‐limiting step in the kynurenine pathway, which enhances immune suppression. Tryptophan serves as a precursor for several neurotransmitters and neurochemicals, including 5‐HT and melatonin. Serotonin (5‐hoxytryptamine, 5‐HT) has been documented to augment cAMP and Ca^2+^ content in cells [30, 31, 32], which play an important role as second messengers [33, 34]. In the brain, serotonin serves as a substrate for *N*‐methyltransferases, enzymes that convert serotonin into *N*‐methyltryptamines. These metabolites have psychoactive properties and can be hallucinogenic in humans [35]. Our metabolomic analysis revealed an elevation in three metabolites (i.e., L‐kynurenine, 3‐hydroxyanthranilate and tryptamine) and a reduction in two metabolites (i.e., 5‐Hydroxy‐L‐Tryptophan and 5‐HT) (Figure 6B–F). Moreover, the expression of INMT, KMO, AADAT, CAT and NMNAT3 were all increased in the cumulus cells from old mice (Figure 6G–J). These observations suggest an enhanced metabolism of tryptophan through the kynurenine pathway in aged cumulus cells.

**FIGURE 7:**
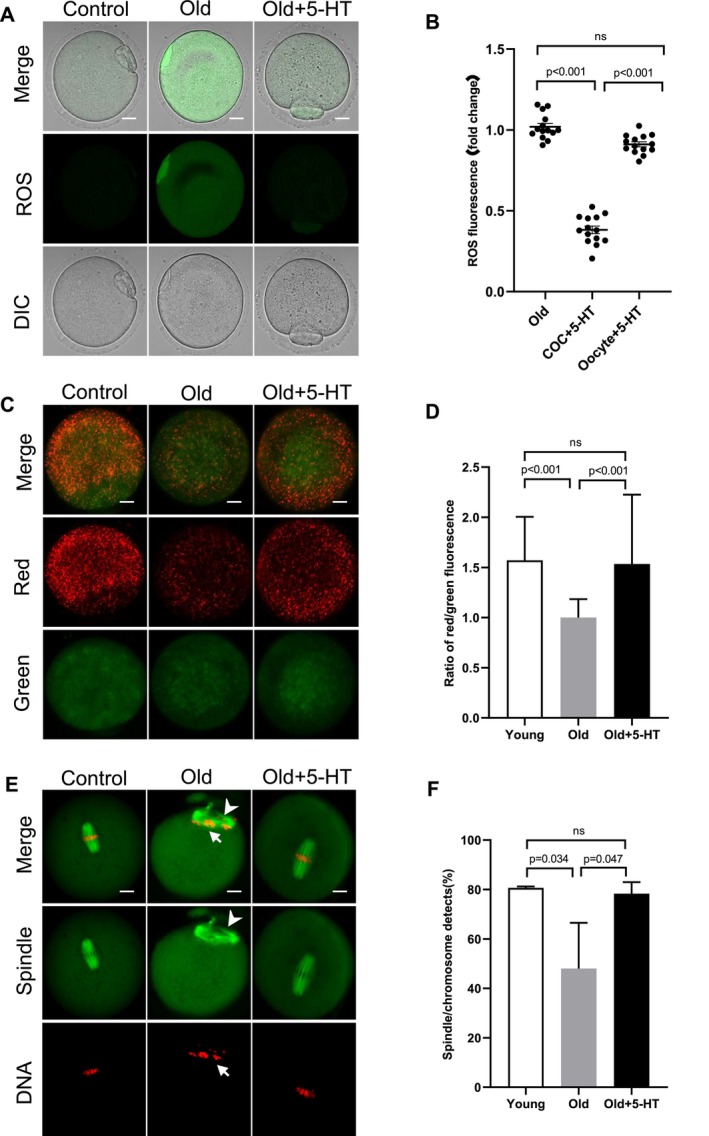
Effects of in vitro supplementation of 5‐HT on quality of old oocytes. (A) Representative images of CM‐H2DCFAD (green) fluorescence in young, old and old + 5‐HT oocytes. (B) Quantification of the relative ROS level (*n* = 14 for each group). (C) Mitochondrial membrane potential in young, old and old + 5‐HT oocytes were assessed by JC‐1 staining. The green fluorescence shows the inactive mitochondria and the red fluorescence shows the active mitochondria in oocytes (D) Histogram showing the JC‐1 red/green fluorescence ratio (at least 30 oocytes for each group) (E) Representative examples of meiotic spindle and chromosomes in indicated oocytes. Spindle disorganisation and chromosome misalignment are indicated by arrowheads and arrows, respectively. (F) Quantitative analysis of meiotic defects in indicated oocytes. At least 40 oocytes for each group were analysed, and the experiments were conducted three times. Data are expressed as the mean ± SD from three independent experiments. Scale bars, 15  μm.

Since the decreased 5‐HT in aged cumulus cells, we determined whether the addition of 5‐HT could correspondingly rescue the phenotypes of aged oocytes. We divided our study into three groups: young group, old group and old oocytes treated with 5‐HT (old + 5‐HT) group. Briefly, COCs from old mice were cultured in medium supplemented with 5‐HT, and then the relevant oocyte phenotypes were subsequently evaluated. The chosen 5‐HT dose (0.4 μg/mL) was based on the published literature [36]. Reactive oxygen species (ROS) accumulation in metaphase II (MII) oocytes from young and old mice was monitored by staining with 5‐(and‐6)‐chloromethyl‐2′,7′‐dichlorodihydrofluorescein diacetate (CM‐H2DCFDA) fluorescent dye. As shown in Figure 7A,B, the quantitative analysis revealed a significant upregulation of signals in aged oocytes compared to young oocytes, indicative of the higher ROS levels, which is consistent with the published data [37]. Remarkably, we found that the level of ROS from the old + 5‐HT group was significantly reduced compared to the old group. Given the common association between mitochondrial function and oxidative stress, we further assessed whether the addition of 5‐HT influenced the mitochondrial membrane potential (ΔΨ), utilising JC‐1 staining in old oocytes. The fluorescent dye JC‐1 undergoes a shift from green to red with increasing ΔΨ. As illustrated in Figure 7C,D, there was a reduction in ΔΨ in old oocytes compared to young oocytes, and notably, the supplementation of 5‐HT significantly prevented this decline. Lastly, previous studies have shown that aging results in spindle and chromosomal abnormalities in oocytes [38]. To visualise the spindle, three groups were immunolabelled with anti‐ɑ‐tubulin antibody. Simultaneously, propidium iodide staining was employed for chromosome visualisation. The quantitative analysis presented in Figure 7E,F revealed that 80% of young oocytes exhibited a typical barrel‐shaped spindle with well‐aligned chromosomes on the equator. In contrast, 52% of oocytes obtained from the old group showed a disorganised spindle or misaligned chromosomes. Intriguingly, these defects were detected in only 22% of aged oocytes administered with 5‐HT. In conclusion, these results suggest that in vitro administration of 5‐HT ameliorates both metabolic dysfunction and meiotic defects in aged oocytes.

### 2.4 Nucleotide Metabolism and Carbohydrate Metabolism in Aged Cumulus Cells

**FIGURE 8:**
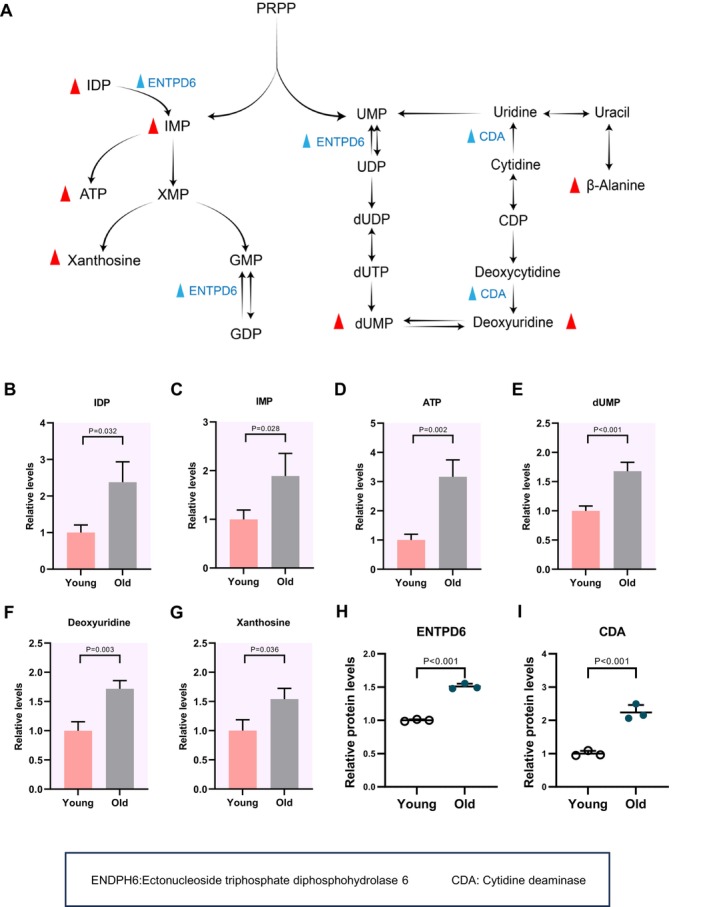
Increased nucleotide metabolism in aged cumulus cells (A) Schematic diagram of nucleotide metabolism. Increased metabolites in cumulus cells in aging process are indicated by bold red triangles. Differential metabolic enzymes changes are indicated by blue triangles. (B–G) Relative levels of metabolites related to nucleotide metabolism in cumulus cells at different ages. (H and I) Relative abundance of the representative enzymes involved in nucleotide metabolism. Error bars, SEM. Student's *t*‐test was used for statistical analysis in all panels, comparing to young group cumulus cells. n.s., not significant.

Purines and pyrimidines are pivotal components in developmental processes, serving as precursors for some cofactors and nucleic acids, or functioning as an energy carrier [39]. The de novo purine biosynthesis initiates with phosphoribosyl pyrophosphate and culminates in IMP (Figure 8A). Metabolome profiles revealed the increased levels of IDP, IMP, ATP, xanthosine, dUMP and deoxyuridine in cumulus cells from old mice compared to cells from young mice (Figure 8B–G). In support of this, an integrated analysis of proteomics coupled with metabolic pathways identified the elevation of two enzymes (ENTPD6 and CDA) in aged cumulus cells (Figure 8H,I). These results indicate the elevated activity of nucleotide metabolism in cumulus cells from old mice.

In addition, the tricarboxylic acid (TCA) cycle stands as a vital metabolic pathway, providing the energy for cellular activities [40]. In cumulus cells of old mice, three differential metabolites (i.e., malate, 2‐oxoglutarate and pyruvate) exhibited upregulation (Figure S3A–E). The proteomic profiles identified the elevated accumulation of 2 enzymes (PCX and PDHB) in the TCA cycle (Figure S3F,G). Collectively, these findings indicate that the active TCA and nucleotide metabolism in aged cumulus cells may serve as a compensatory mechanism for energy provision to the surrounded oocytes.

## 3 Discussion

The status and events of intracellular metabolism can be reflected through metabolomics. However, profiling of metabolites in cumulus cells from old mice is still lacking. Here, we performed metabolomic and proteomic analyses of mouse cumulus cells to elucidate the effects of aging on their metabolic characteristics. Through multi‐omics analysis, we found some significant changes in aged cumulus cells, including (1) enhanced fatty acid beta‐oxidation, (2) increased arginine and lysine catabolism, (3) elevated degradation in BCAAs, (4) active tryptophan metabolism.

As hydrophobic or amphipathic molecules, lipids play crucial roles in various biological events, serving as energy sources, mediators of cell signalling, and essential components of membranes in both organelles and the plasma membrane. However, high concentrations of fatty acids can negatively impact oocyte quality [41]. In bovine, supplementation of NEFAs (palmitic, stearic and oleic acids) during IVM induces the increased levels of genes associated with energy metabolism and oxidative stress in oocytes, and oocyte‐derived blastocysts exhibit significantly lower cell numbers and increased apoptosis [42, 43]. Herein, we observed the elevated fatty acid β‐oxidation with high concentrations of fatty acids in cumulus cells. Such a signature may represent a connection between oocyte quality and reproductive aging.

Intricate metabolic pathways are demanded in order to catch an adequate energy supplement, including fatty acid metabolism, carbohydrate metabolism and amino acid metabolism. Enhanced fatty acid beta‐oxidation strongly indicates that lipid metabolism plays an important role in energy provision for cumulus cells from old mice. Studies have shown that carbohydrate metabolism could be inhibited by higher lipid metabolism [44]. This result may be explained by the fact that energy production by lipid metabolism cannot meet the physiological needs of cumulus cells or oocytes in old mice, and amino acids serve as oxidisable substrates for energy supply. There may be four reasons for the energy gap: (1) abnormal mitochondrial rate in aged cumulus cells was elevated; (2) increased energy requirements to maintain normal physiological status in cumulus cells affected by aging; (3) reduced activity of enzymes involved in energy supply‐related reactions; (4) occurrence of energy barriers in old cumulus cells. Lu et al. have demonstrated that maternal aging negatively influences the mitochondrial activity of cumulus cells, leading to mitochondrial morphology and structure alterations, and decreased levels in deoxyribonucleic acid copy number and adenosine triphosphate [45]. Our proteomics results showed an overall upward trend, and increased fatty acid content may provide a safeguard for energy. Nevertheless, excessive fatty acid may have adverse effects on oocyte quality [44], potentially contributing to higher ROS accumulation in the old group compared to the young group. On this basis, our study revealed a significant increase in solute carrier family 2 member 2 (SLC2A2, also known as GLUT2) accumulation in cumulus cells obtained from old mice. This accumulation was found to enhance the uptake of glucose and fructose [46, 47]. However, no differential metabolites related to these processes were detected in aged mice. Two questions remain unanswered for future investigation: why the accumulation of GLUT2 does not increase the content of glucose, and whether upregulated glucose metabolism can reduce the adverse effects of aging.

Metabolism of amino acids has been implicated in follicular and oocyte development. For example, in bovine, the addition of L‐arginine increased the integrity of cumulus cells exhibiting plasma membrane after 22 h of culture [27]. In primiparous lactating sows, a low intake of lysine negatively affected follicular development and diminished the capacity of follicles to support oocyte maturation. When a standardised pool of oocytes was cultured with follicular fluid collected from the follicles of sows on a low lysine diet, fewer oocytes reached MII of meiosis [48]. Studies suggest that during metabolic stress, BCAA catabolism enhances lipogenesis and competes with lipid oxidation, resulting in the accumulation of toxic lipid intermediates, and removing BCAAs from myocyte culture media inhibited ATP synthase‐mediated lipid droplet formation [49, 50]. It is reasonable to hypothesise that the negative effects of aging on oocytes are related to the disturbance of amino acid metabolism in cumulus cells. As an essential amino acid in the human body, tryptophan plays various roles. One of its important functions is serving as a precursor for several neurotransmitters and neurochemicals, including 5‐HT and melatonin. 5‐HT, a metabolite derived from tryptophan, plays a critical role in multiple biological processes. Serotonin halts meiosis by acting on the 5‐HT7 receptor, which is coupled to the Gs‐protein. This interaction stimulates the adenylate cyclase system of second messengers. Although the molecular mechanisms remain unclear, 5‐HT seems to be able to regulate oocyte maturation in various species, like molluscs, crustaceans, nemerteans, fish and mammals [36]. Besides, the level of 5‐HT uptake by oocyte has been correlated with granulosa proliferation and follicle size [36]. Therefore, while elevated 5‐HT levels are imperative for oocyte maturation in aged mice, they are also perhaps essential for sustaining physiological functions in cumulus cells. In support of this conception, here we found that supplementation of 5‐HT significantly improves the oocyte quality from old mice (Figure 7). In addition, enhanced activation of the kynurenine pathway may directly compromise mitochondrial function by increasing the production of ROS and 3‐hydroxykynurenine [51]. Alterations in tryptophan metabolism, as observed, provide valuable insights for the prediction and improvement of oocyte quality from old mice.

In our study, we performed metabolomic and proteomic profiling of cumulus cells from young and old mice and elaborated on the dynamic changes in metabolic pathways in aged cumulus cells. These findings not only reveal the potential metabolic networks controlling oocyte development but also may facilitate the discovery of potential molecular biomarkers that could be used to predict and improve oocyte quality. Previous studies have demonstrated the significant differences in the arginine catabolic pathway in both oocytes and cumulus cells from sow and goat during maturation, indicative of its crucial role for oocyte development [52, 53, 54]. Xiong et al. [9] found that the exogenous supplementation of polyamine spermidine, a product of the arginine catabolic pathway, could attenuate the age‐induced meiotic defects of oocytes. Interestingly, we observed the significantly decreased level of phosphocreatine in the arginine catabolic pathway in aged cumulus cells. Therefore, the metabolic intervention of metabolites from the arginine catabolic pathway to aged oocytes, such as phosphocreatine, deserves further exploration. However, there are some potential limitations in our study. First, we focused only on describing changes in the levels of differential metabolites. Non‐differential metabolites such as melatonin, progesterone, and nicotinamide adenine dinucleotide may also have the potential to ameliorate the effects of aging on cumulus cells and oocytes, and their roles should be explored in future studies. Second, although the expression of many proteins was not changed in old cumulus cells, it is still possible that they may function in metabolic activity through the regulation of post‐translational modifications and structural alterations. Further investigation is needed to explore this possibility. Finally, in the present study, we evaluated the effect of 5‐HT on oocyte quality, which may act on cumulus cells, oocytes or both, and the conversion between research findings and clinical application still remains a huge challenge. However, due to limitations in the oocyte culture system, we were unable to dissect the specific interactions between 5‐HT and these cellular components.

## 4 Materials and Methods

### 4.1 Mice

All experiments strictly adhered to the guidelines set forth by the Animal Care and Use Committee of Nanjing Medical University, with prior approval obtained (IACVC: 1703017). Female C57BL/6 mice were categorised into two groups based on age: the young group (8 weeks old) and the old group (42 weeks old, nearing their terminal generative lifespan). The experimental mice were individually housed in ventilated cages, with each cage accommodating up to five mice. All mice were maintained under constant conditions, including a temperature of 22°C, specified pathogen‐free environments, and a 12‐h light/12‐h dark cycle. Uniform and unrestricted access to food and water was provided to all mice. Euthanasia was performed prior to ovary collection, utilising cervical dislocation following carbon dioxide‐induced narcosis.

### 4.2 Cumulus Cells Collection

Female mice underwent initial injection with 5 units of pregnant mare serum gonadotropin (PMSG), followed by a subsequent injection of 5 units of human chorionic gonadotropin (hCG) 48 h later to induce ovulation. Euthanasia was carried out via cervical dislocation approximately 14 h after the hCG injection. For the collection of cumulus surrounding MII oocytes, COCs were isolated from the ampullary region of the oviducts. These complexes were then placed into M16 medium (Nanjing Luanchuang Co., China) containing 0.5 mg/mL hyaluronidase at 37°C to facilitate the release of cumulus cells. Ten sets of samples were meticulously collected for both young and old groups, with 2.4 × 10^5^ cumulus cells separated from 200 MII COCs per sample for metabolomics and 4.5 × 10^5^ cumulus cells per sample in each of the three biological replicates for proteomics.

### 4.3 Proteomics

As previously detailed, proteome data collection and analysis were meticulously conducted [55]. Cumulus cells from the young and old mice were lysed in protein extraction buffer including 8 M urea, 75 mM NaCl, 50 mM Tris, pH 8.2, 1% (v/v) EDTA‐free protease inhibitor, 1 mM NaF, 1 mM b‐glycerophosphate, 1 mM sodium orthovanadate, 10 mM sodium pyrophosphate and 1 mM phenylmethylsulfonyl fluoride (PMSF). The extracted proteins underwent reduction, alkylation and digestion as described previously [56]. Following digestion, the resulting peptides were subjected to the TMT‐6plex labelling according to the manufacturer's protocols. The mixed TMT‐labelled peptides underwent separation using the high‐pH reversed phase (HP‐RP) fractionation technology based on the ACQUITYUPLC M‐class system (Waters) with a BEH C18 Column (300 μm × 150 mm, 1.7 μm; Waters) to generate a total of 30 fractions. The resulting fractions were freeze‐dried after collection. All fractions were sequentially reconstituted in 0.1% formic acid (FA) and analysed using an Orbitrap Fusion Lumos mass spectrometer (ThermoFisher Scientific) coupled to a Proxeon EasynLC 1200 system. Peptides were separated with an analytical column (75 μm × 150 mm, 1.7 μm, CoAnn Technologies) using a 95 min linear gradient (3% to 5% buffer B for 5 s, 5% to 15% buffer B for 40 min, 15% to 28% buffer B for 34 min and 50 s, 28% to 38% buffer B for 12 min, 38% to 100% buffer B for 5 s, 100% buffer B for 8 min) at 300 nL/min. The MS parameters refer to previously published [57]. Briefly, data were acquired with an MS1 scan for a *m*/*z* range 350–1500 with a resolution of 60,000 followed by data dependent HCD MS2 spectra in the Orbitrap with a resolution of 15,000 and HCD collision energy of 36%. All raw files were analysed using MaxQuant software (V1.6.5.0) against the UniProt mouse proteome database [58]. Trypsin/P enzyme specificity was selected, allowing up to two missed cleavages. Fixed modifications included Carbamidomethylation of cysteine residues (+57.0215 Da), with methionine oxidation and protein N‐terminus acetylation were set as variable modifications. The TMT reporter ion MS2 method of isobaric labels in MaxQuant was set for protein quantification. Peptides and proteins with FDR threshold < 0.01 having at least one unique peptide were considered confident. Statistical significance in protein abundance while between two groups was determined by *p* value < 0.05 (Student's *t*‐test) and fold change > 1.5. A dendrogram with heatmap was produced to illustrate the extent of similarity of protein expression among the samples using the package of ComplexHeatmap in RStudio. Ensembl gene IDs from differentially expressed proteins were utilised for subsequent bioinformatics analysis and underwent principal component analysis (R package: FactoMineR). KEGG pathways were employed for enrichment analysis by using the clusterProfiler R package. The flowchart of metabolomics and proteomics was adapted from Servier Medical Art.

### 4.4 Metabolomics

The cumulus cell samples were transferred to Eppendorf (EP) tubes and promptly flash‐frozen in liquid nitrogen, subsequently stored at −80°C for future use. For experimental purposes, all samples were resuspended in 300 μL of 80% methanol/water (vol/vol) and homogenised using an Ultra‐Turrax homogeniser. Following centrifugation at 16,000*g*, 4°C for 15 min, with a preceding 10‐min cooling period on ice, the supernatant was transferred into new tubes, and dried samples were stored at −80°C for subsequent analysis. The standard metabolic profiling method we used has been published [56]. Concisely, the instrumental analysis was performed on a UPLC Ultimate 3000 system (Dionex, Germering, Germany), coupled to a Q‐Exactive mass spectrometer (Thermo Fisher Scientific, Bremen, Germany). In order to avert complications caused by the injection order, all samples were analysed in a random fashion. Chromatographic separation was achieved by using a Hypersil GOLD C18 column (100 mm × 2.1 mm, 1.9 μm) from Thermo Fisher Scientific, with the temperature set at 40°C and a flow rate of 0.4 mL/min. Two mobile phases were used: pure ACN containing 0.1% FA as phase A, and 0.1% FA in ultrapure water as phase B. Initially, 1% (vol/vol) phase A eluted for 3 min, and the percentage of phase A gradually increased to 99% (vol/vol) (*t* = 10 min), followed by a 5‐min 99% (vol/vol) phase A elution (*t* = 15 min). Finally, the percentage of phase A immediately reduced to 1% (vol/vol) and was maintained for 2 min (*t* = 17 min). The mass spectrometer was operated in a full‐scan mode within the range from 70 to 1050 *m*/*z*, employing a resolution of 70,000 in both positive and negative modes. The TraceFinder v5.1 software from Thermo Fisher Scientific was utilised to process the raw data obtained from the mass spectrometer. The identification of metabolites was accomplished by comparing accurate mass and retention time with commercially available standard compounds, utilising the author‐constructed library. Statistical analyses were performed using “R” (V4.1.0). Student's *t*‐test was employed to compare continuous variables between two groups [59]. OPLS‐DA analysis was carried out using SIMCA‐P software (V14.1; Umetrics AB, Umea, Sweden). A combined threshold for statistical significance was applied, considering both the VIP value (> 1.00) and a *p* value (< 0.05) for each metabolite. In order to integrate metabolomics and proteomics data, the KEGG Mapper (V4.1) was used for this purpose.

### 4.5 Collection and Culture of Oocytes

COCs were obtained 48 h after PMSG injection from two distinct groups. COCs obtained from mice aged 8 weeks were cultured with normal M16 medium, categorising them as the young group. For mice aged 42 weeks, they were randomly divided into two subgroups. One was cultured with M16 medium, designated as the old group, while the other, after 1 h, was cultured with the same medium containing 0.4 μg/mL 5‐HT (H7752 Merck KGaA, Darmstadt, Germany) and was termed the save group. All COCs underwent cultivation in a humid environment at 37°C with 5% CO_2_. After a 12–14 h incubation period, cumulus cells were carefully removed by repeated pipetting, resulting in the acquisition of denuded oocytes.

### 4.6 Immunofluorescence Staining

Obtained MII oocytes were fixed with 4% paraformaldehyde (281692, Santa Cruz) for 30 min, followed by permeabilisation with 0.5% Triton X‐100 (T8200, Solarbio) for 15 min at room temperature. All oocytes underwent washing in PBS (SH30256.01B, Thermo) supplemented with 1% bovine serum albumin (A2153, Sigma) three times and were blocked for 1 h, then incubated overnight at 4°C with primary antibody (FITC‐α‐tubulin, F‐2168, Sigma‐Aldrich). The nuclei were stained with Propidium Iodide (red, P4170, Sigma) for 10 min after washing three times. Oocytes were mounted on anti‐fade (P1026, Beyotime Biotechnology) medium and imaged under the laser scanning confocal microscope (LSM 710, Zeiss, Germany) after washing three times ImageJ (NIH) was used for multiple images processing and intensity measurements, and the data were normalised concerning background levels.

### 4.7 JC‐1 Staining

A concentration of 10 μg/mL JC‐1 (Tetrachloro‐tetraethyl benzimidazol carbocyanine iodide) was utilised to stain the MII oocytes for 25 min. Subsequently, PBS was employed to wash the oocytes three times. No more than 15 oocytes were then transferred to a live cell‐imaging dish covered with paraffin. Imaging was conducted using a laser scanning confocal microscope. The fluorescence intensity of red and green light was measured by enzyme‐labelling instrument. The ratio of red/green fluorescence intensity indicates membrane potential. The excitation wavelength for red light is 559 nm, and the emission wavelength is 572 nm. For green light, the excitation wavelength is 488 nm, and the emission wavelength is 520 nm [36].

### 4.8 ROS Evaluation

The M16 medium, enriched with 5 μM CM‐H2DCFDA (C6827, Invitrogen), was employed for incubating oocytes for a duration of 30 min at 37°C. Following three washes, the oocytes were transferred to a cell‐imaging dish covered with mineral oil and subjected to scanning using a laser scanning confocal microscope from ZEISS.

### 4.9 Statistical Analysis

Unless explicitly mentioned otherwise, each experiment was replicated a minimum of three times for robustness and reliability. Statistical analyses were conducted using GraphPad Prism 8.0.2, presenting the data as means ± SD, with the exception of metabolomics and proteomics data, which were expressed as means ± SEM. A probability value of < 0.05 was considered indicative of statistical significance.

## Author Contributions

L.S., L.G. and Q.W. designed the research. L.S., H.W. and S.Z. performed the research. L.S., M.C. and X.G. analysed the data. L.S., Q.W. and L.G. wrote the paper.

## Ethics Statement

All animal experiments were conducted in strict compliance with the regulations and guidelines established by the local animal ethics committee and approved by the Animal Care and Use Committee of Nanjing Medical University (IACVC: 1703017). This study does not contain human participants.

## Conflicts of Interest

The authors declare no conflicts of interest.

## Supporting information


**Figure S1.** Active glycine, serine and threonine metabolism in aged cumulus cells. (A) Schematic diagram of glycine, serine and threonine metabolism. Increased metabolites in cumulus cells from old mice are indicated by bold red triangles. Changes in differential metabolic enzymes are indicated by blue triangles. (B and C) Relative levels of metabolites related to glycine, serine and threonine metabolism in young and aged cumulus cells. (D–J) Relative abundance of the representative enzymes involved in fatty acid beta oxidation. Error bars, SEM. Student's *t*‐test was used for statistical analysis in all panels, comparing to young cumulus cells. n.s., not significant.

**Figure S2.** Active histidine metabolism in aged cumulus cells (A) Schematic diagram of histidine metabolism. Increased metabolites in cumulus cells from old mice are indicated by bold red triangles. (B–D) Relative levels of metabolites related to histidine metabolism in young and aged cumulus cells. Error bars, SEM. Student's *t*‐test was used for statistical analysis in all panels, comparing to young cumulus cells. n.s., not significant.

**Figure S3.** Increased TCA cycle in aged cumulus cell. (A) Schematic diagram of TCA cycle. Increased metabolites in cumulus cells in aging process are indicated by bold red triangles. Differential metabolic enzymes changes are indicated by blue triangles. (B–E) Relative levels of metabolites related to TCA cycle in cumulus cells at different ages. (F and G) Relative abundance of the representative enzymes involved in TCA cycle. Error bars, SEM. Student's *t*‐test was used for statistical analysis in all panels, comparing to young group cumulus cells. n.s., not significant.

**Table S1.** Proteomics profiling in cumulus cells from young and old mice.

**Table S2.** Metabolomic profiling in cumulus cells from young and old mice.

## Data Availability

The mass spectrometry proteomics data have been deposited in the ProteomeXchange Consortium via the PRIDE [60] partner repository with the dataset identifier PXD050687.
