# Supplementary material for: Multi‐Omics Reveal the Metabolic Changes in Cumulus Cells During Aging
Source: Cell Prolif. 2025 Mar 5;58(8):e70014. doi: 10.1111/cpr.70014 (PMC12336455; doi:10.1111/cpr.70014)
Supplement: Supplementary file 1 — Figure S1. Active glycine, serine and threonine metabolism in aged cumulus cells. (A) Schematic diagram of glycine, serine and threonine metabolism. Increased metabolites in cumulus cells from old mice are indicated by bold red triangles. Changes in differential metabolic enzymes are indicated by blue triangles. (B and C) Relative levels of metabolites related to glycine, serine and threonine metabolism in young and aged cumulus cells. (D–J) Relative abundance of the representative enzymes involved in fatty acid beta oxidation. Error bars, SEM. Student's t‐test was used for statistical analysis in all panels, comparing to young cumulus cells. n.s., not significant. [file CPR-58-e70014-s003.pdf]

Supplementary Fig. 1.

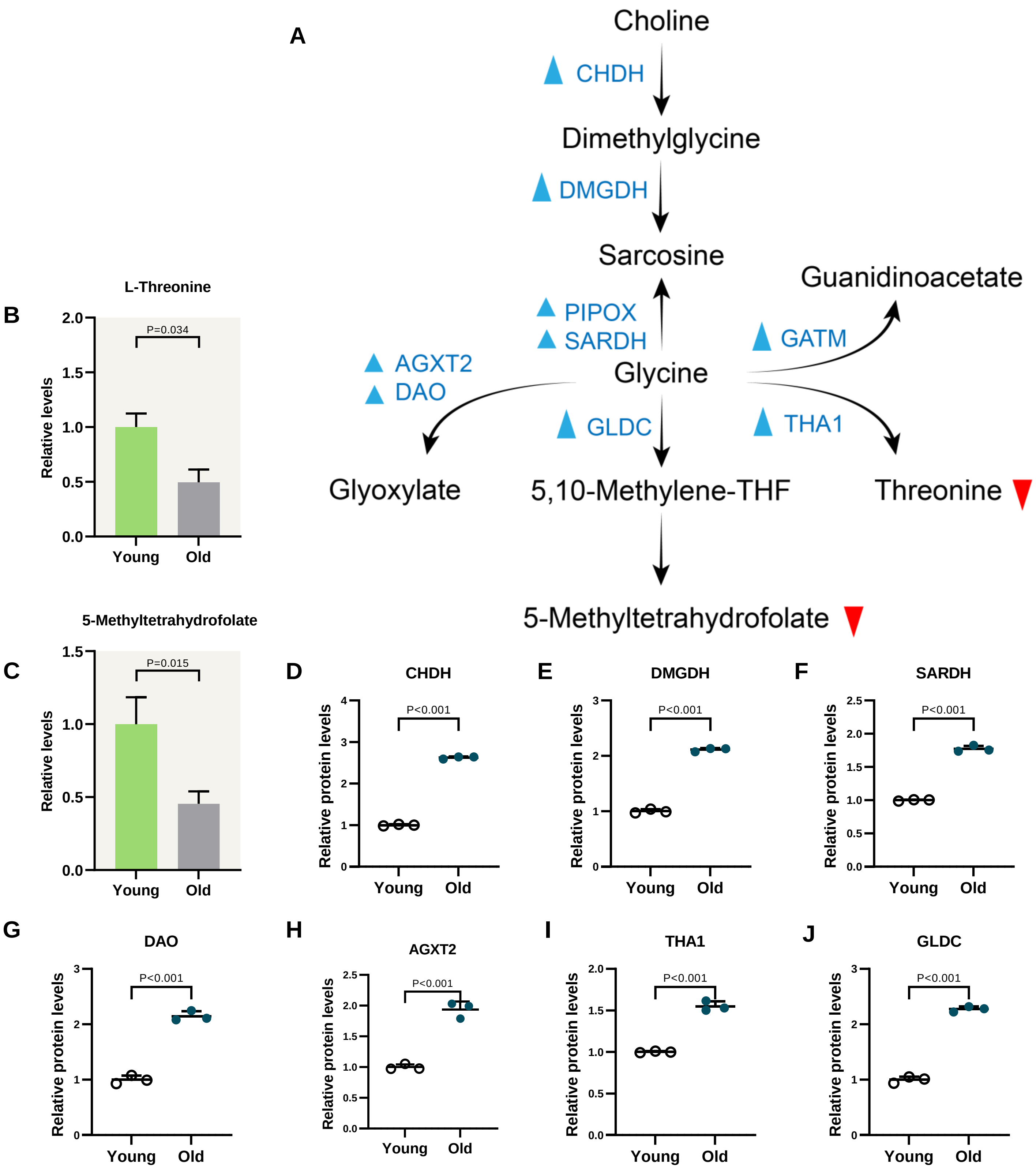

CHDH: Choline dehydrogenase
DMGDH: Dimethylglycine dehydrogenase precursor
SARDH: Sarcosine dehydrogenase
GLDC: Glycine decarboxylase
AGXT2: Alanine-glyoxylate aminotransferase 2
DAO: D-amino acid oxidase
THA1: Threonine aldolase 1
