# Supplementary figures and images for: Multi‐Omics Reveal the Metabolic Changes in Cumulus Cells During Aging
Source: Cell Prolif. 2025 Mar 5;58(8):e70014. doi: 10.1111/cpr.70014 (PMC12336455; doi:10.1111/cpr.70014)

Supplementary Fig. 2.

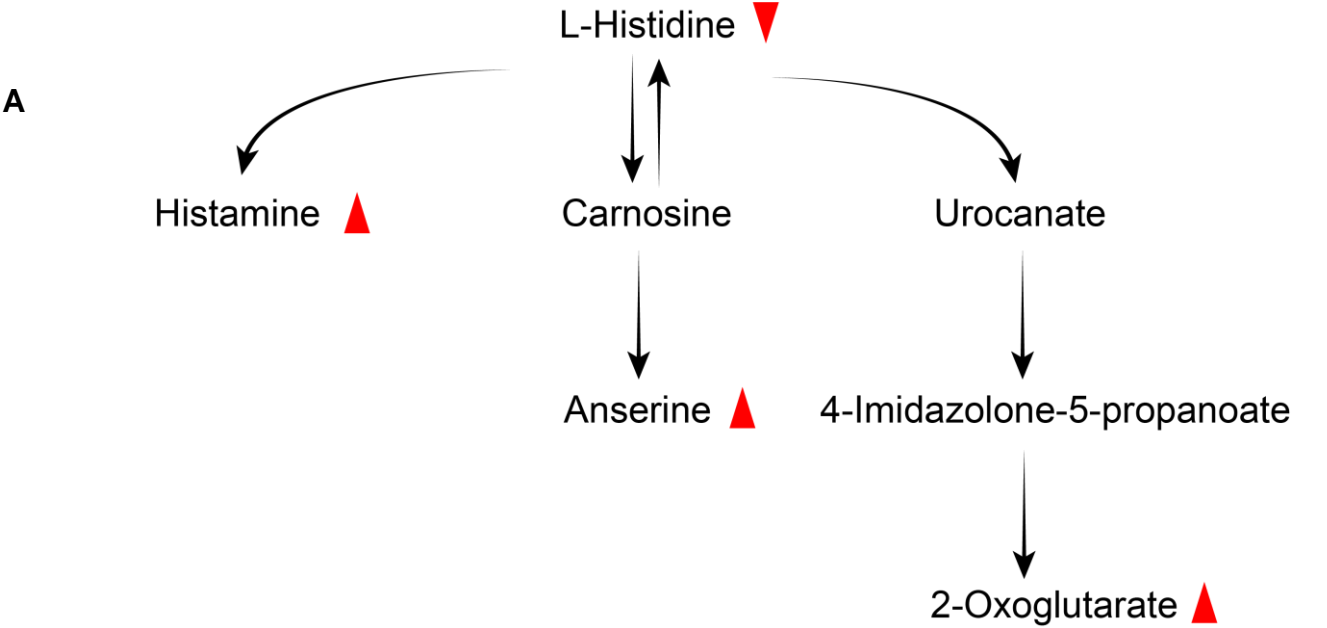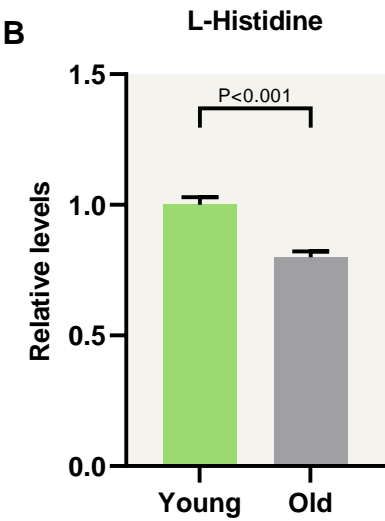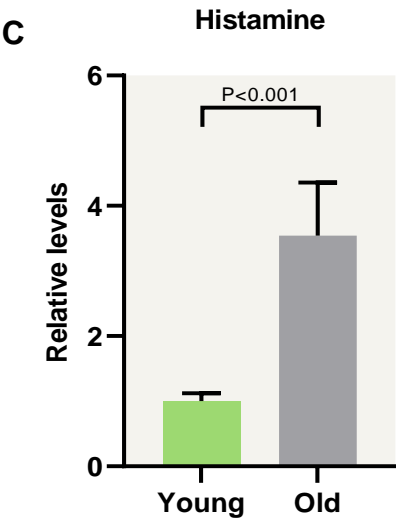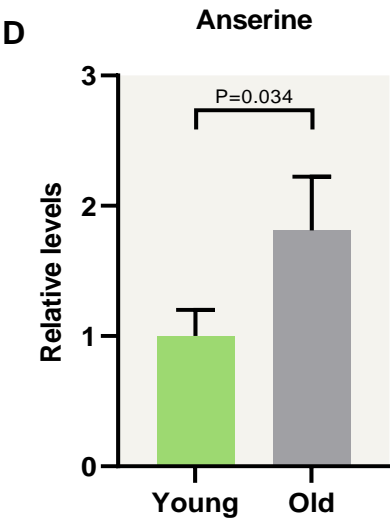

Supplement: Supplementary file 2 — Figure S2. Active histidine metabolism in aged cumulus cells (A) Schematic diagram of histidine metabolism. Increased metabolites in cumulus cells from old mice are indicated by bold red triangles. (B–D) Relative levels of metabolites related to histidine metabolism in young and aged cumulus cells. Error bars, SEM. Student's t‐test was used for statistical analysis in all panels, comparing to young cumulus cells. n.s., not significant. [file CPR-58-e70014-s005.pdf]

Supplementary Fig. 3.

A

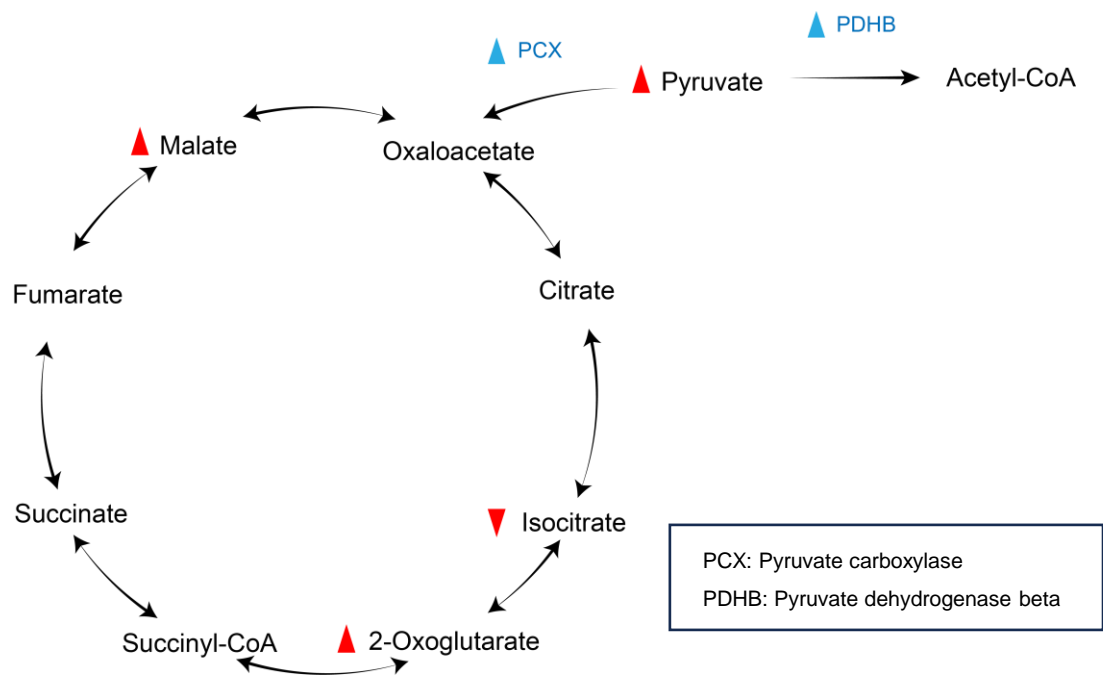

B

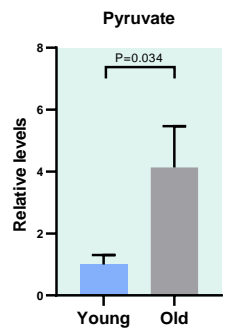

C

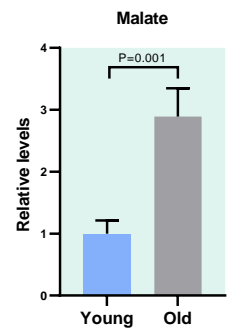

D

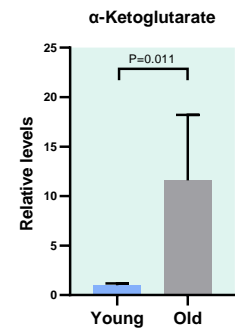

E

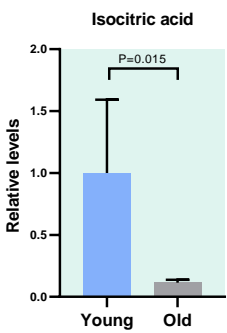

F

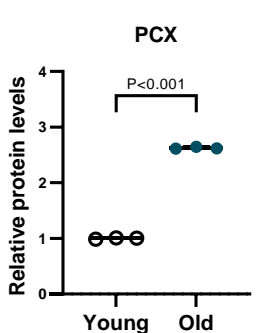

G

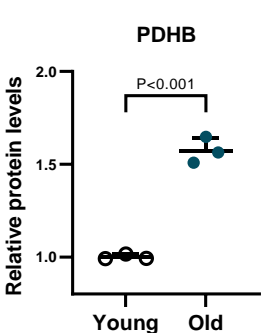

Supplement: Supplementary file 3 — Figure S3. Increased TCA cycle in aged cumulus cell. (A) Schematic diagram of TCA cycle. Increased metabolites in cumulus cells in aging process are indicated by bold red triangles. Differential metabolic enzymes changes are indicated by blue triangles. (B–E) Relative levels of metabolites related to TCA cycle in cumulus cells at different ages. (F and G) Relative abundance of the representative enzymes involved in TCA cycle. Error bars, SEM. Student's t‐test was used for statistical analysis in all panels, comparing to young group cumulus cells. n.s., not significant. [file CPR-58-e70014-s002.pdf]
